# Effectiveness and safety of Chinese herbal medicine Xiaoyao san for the treatment of insomnia: Protocol for a systematic review and meta-analysis

**DOI:** 10.1097/MD.0000000000016481

**Published:** 2019-07-19

**Authors:** Jiamin Li, Zhenni Mu, Jun Xie, Tongtong Ma, Ying Zhang, Xianghua Qi, Jing Teng

**Affiliations:** a Shandong University of Traditional Chinese Medicine; b Affiliated Hospital of Shandong University of Traditional Chinese Medicine, China.

**Keywords:** Chinese herbal medicine, insomnia, protocol, systematic review, Xiaoyao san

## Abstract

**Background:**

Insomnia is one of the most common sleep problems, which can impact physical and mental quality of life, resulting in a heavy social and economic burden. Xiaoyao san, a Chinese Herbal Medicine product, has been widely used as an alternative to recommended treatments for insomnia, but still lack of evidence of evidence-based medicine, which the clinical efficacy and its safety are questioned. Accordingly, we provide a protocol to evaluate the efficacy and safety of Xiaoyao san to update the search and evaluation for the best available and security treatment for insomnia.

**Methods:**

This review systematic and comprehensive retrieves of 8 related databases at home and abroad. Only randomized controlled trials (RCTs) of Xiaoyao san on Insomnia published in English and Chinese will be included. The quality of the included trials including randomization, allocation concealment, blinding, withdrawal, and loss of follow-up which was evaluated using internationally accepted evaluation criteria. And then systematically comprehensive analysis of the efficacy.

**Results:**

This review will be to assess the efficacy and safety of Xiaoyao san for insomnia.

**Conclusion:**

This systematic review will provide strong evidence for the effectiveness and safety of Xiaoyao san in the treatment of insomnia.

**PROSPERO registration number:**

CRD42019127326

## 1 Introduction

Insomnia refers to the subjective perception of difficulty with sleep initiation, duration, consolidation, or quality that occurs despite adequate opportunity for sleep, and that results in some form of daytime impairment.^[1]^ Insomnia is one of the most common sleep problems that occurs in individuals of all ages^[2,3]^ and has become a global public health problem.^[4]^ Epidemiological studies have shown that one-third of adults in Western countries experience difficulty with sleep initiation or maintenance at least once a week, and 6% to 15% Can be diagnosed as a patient with insomnia.^[5]^ 45.4% of respondents in China have experienced varying degrees of insomnia in the past month.^[6]^ Insomnia not only impairs human health, but also has socio-professional consequences,^[7]^ which brings heavy pressure and economic burden to families and society.^[8]^

Modern medical interventions for insomnia include psychotherapy, drug therapy, and physical therapy. However, because psychotherapy can be implemented poorly in the clinic, the efficacy of physical therapy remains to be verified, and drug treatment has problems such as drug dependence and addiction. The treatment of insomnia still needs further exploration and improvement. Insomnia is called “bu mei” in ancient China. Its pathogenesis is directly related to the yin and yang, viscera and bowels, qi-blood disorders of the human body, and the underlying cause can be traced back to human personality and physique aiming at its root causes. In the treatment of insomnia, traditional Chinese medicine pays more attention to the analysis in different dimensions of space and time, and pays more attention to the influence of abnormal factors in the natural environment, social and humanistic environment, human's own mental and physical environment on human sleep at different times.

Xiaoyao san is a Chinese medicine prescription invented based on the theory of traditional Chinese medicine, which is widely used in the treatment of insomnia in China,^[9–12]^ but lacks evidence of evidence-based medicine. At the same time, the clinical efficacy and safety of Xiaoyao san are questioned. Therefore, it is important to update the search and evaluation to provide the best available evidence for insomnia. Here, we provide a protocol to evaluate the efficacy and safety of Xiaoyao san for insomnia.

## 2 Methods

### 2.1 Protocol and registration

The study protocol has been registered on international prospective register of systematic review (PROSPERO). The trial registration number of PROSPERO is CRD 42019127326.

### 2.2 Ethic approval

No ethical issues are foreseen because this study does not involve clinical trials. The research data is from published papers. The results will be submitted to a peer-reviewed journal.

### 2.3 Inclusion criteria for study selection

#### 2.3.1 Types of studies

Only included in the clinical randomized controlled trial (RCT) of Chinese medicine Xiaoyao san and placebo and western medicine for insomnia, the language is not limited.

#### 2.3.2 Types of patients

Patients with a clear diagnosis of insomnia meet the diagnostic criteria for insomnia (the diagnostic criteria for traditional Chinese medicine and Western medicine), and the age and gender of the patients are not limited.

#### 2.3.3 Types of interventions

The intervention of the experimental group was treated with Xiaoyao san alone or with combined Western medicine; the control group was treated with placebo or Western medicine.

#### 2.3.4 Types of outcome measures

##### 2.3.4.1 Primary outcomes

Treatment for 1 course (1 month), to determine the efficacy. Refer to“Diagnostic Efficacy Standards for TCM Syndrome”.^[13]^ Healed: Symptoms and characteristics disappeared, and sleeptime ≥6 hours. Significantly effective: Symptoms and characteristics were significantly improved, and the sleep time increased by >3 hours compared with before treatment. Effective: Symptoms and characteristics have improved, and the increase in sleep time is <3 hours compared with before treatment. Invalid: no improvement in symptoms and characteristics, no increase in sleep time or increased insomnia time.

##### 2.3.4.2 Secondary outcomes

Pittsburgh Sleep Quality Index score: it is widely used in clinical evaluation of insomnia. It is characterized by the organic combination of the quality and quantity of sleep to assess, indicators include patients’ sleep quality, sleep time, sleep efficiency, sleep disorders, hypnotics, and daytime functions. The total effective rate was calculated based on the Pittsburgh Sleep Quality Index (PSQI) reductive ratio.

- ① reduction rate = [(pre-treatment total score-total score after treatment)/total score before treatment] × 100%
- ② efficacy evaluation criteria
- Clinical recovery: 75% ≤ reductive ratio ≤ 100%
- Markedly effective: 50% ≤ reductive ratio < 75%
- Effective: 25% ≤ reductive ratio < 50%
- Invalid: reductive ratio < 25%

### 2.4 Search methods for the identification of studies

The database for electronic retrieval includes: Cochrane Library, PubMed, EMBASE, SinoMed, WOS, CNKI, VIP Data, and Wangfang Data from their inception to March 2019, using a combined text word and heading search strategy. Search for a randomized controlled trial of Xiaoyao san for the treatment of insomnia. No limit to the language type. Xiaoyao san and Insomnia will be the search terms.

#### 2.4.1 Searching other resources

We will manually search for relevant conference papers, journal references, and magazines without an electronic version to identify any grey literature. In addition, we will contact relevant experts to understand their research topics.

### 2.5 Data collection and analysis

#### 2.5.1 Selection of studies

**Figure 1:**
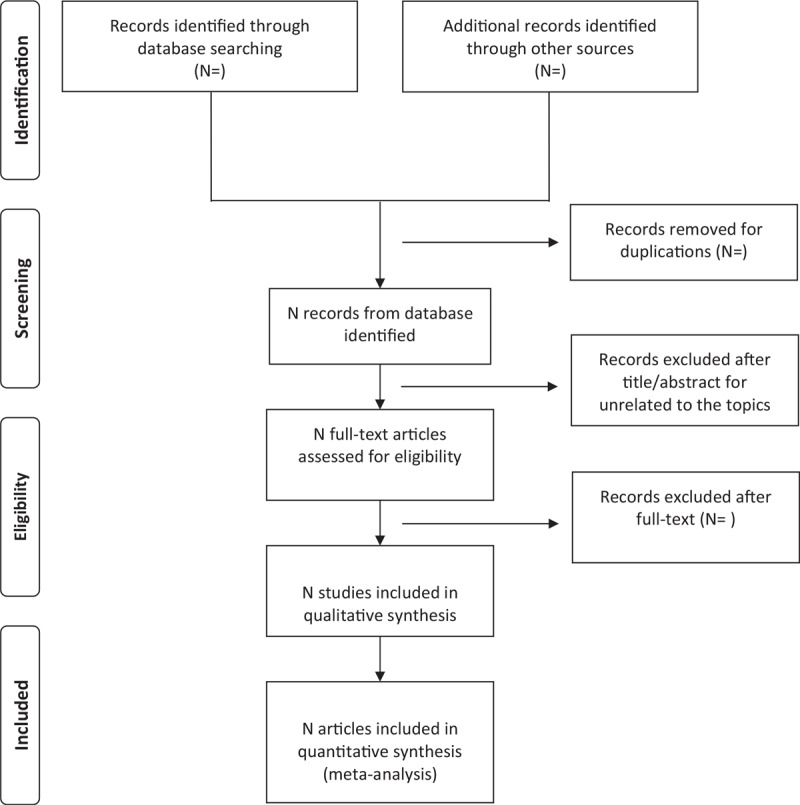
Flow diagram of study selectioNprocess.

Two researchers independently screened and extracted the data according to the inclusion criteria. Researchers extracted the results and examined each other and discussed the inconsistencies in the data screening and extraction process. If there is a difference, it will be arbitrated by JL. For cases where the data is incomplete, we will contact the author to improve. The process of studies selection is presented in a Preferred Reporting Items for Systematic Review and Meta-analysis (PRISMA) flow diagram (Fig. 1).

#### 2.5.2 Data collection and management

Two investigators extracted the following data separately: randomized method, random concealment, sample size, number of cases in each group, age, gender, condition, diagnostic criteria, blindness, blinded subjects, experimental group and control group interventions, follow-up, outcomes, and adverse events.

#### 2.5.3 Assessment of risk of bias in included studies

Two researchers, according to the“risk of bias assessment tool”of the Cochrane Handbook, evaluated the methodological treatment of the included literature. Quality will be evaluated using the following: random sequence generation, random allocation concealment, blind method implementation, outcome assessor, result data integrity, selective outcome report, and other biases. If there are differences, it will be arbitrated by JL.

#### 2.5.4 Measures of treatment effect

For dichotomous data, relative risk with 95% confidence intervals will be presented. For continuous data, mean difference, or standardized mean difference with 95% confidence intervals will be presented.

#### 2.5.5 Dealing with missing data

If the literature data being searched is incomplete or ambiguous, the researcher will contact the author via email or telephone to refine the literature data. If necessary, the articles will be excluded.

#### 2.5.6 Assessment of heterogeneity

A random effect model was used when there was significant heterogeneity between trials (defined as *P* < .1), whereas a fixed effect model was used, and the *I*^2^ test would be used to assess the heterogeneity of the included studies. *I*^2^ > 50 would be considered high. Indicator of heterogeneity. When heterogeneity is observed, we will perform a subgroup analysis to explore possible causes.^[14]^

#### 2.5.7 Assessment of reporting bias

If there is enough research (at least 10 studies), we will use the symmetry of the RevMan funnel plot to determine heterogeneity. However, the asymmetry of the funnel plot does not necessarily mean publication bias, we will try to determine the possibility of any asymmetry. The result will be explained cautiously.

#### 2.5.8 Data synthesis

Statistical analyses will be conducted with RevMan 5.3 software provided by Cochrane Collaboration. For the same unit, the weighted mean differences and the 95% CI analysis were used for the continuous data, and for different units, the standardized mean difference and 95% CI were used. Researchers will evaluate the heterogeneity of the included literatures according to the *I*^2^ tests. If there was statistical homogeneity (*P* > .1, *I*^2^ ≤ 50%) among the research results, then the fixed-effect model was used for the meta-analysis. Otherwise, the data is merged using a fixed effect model. For materials that cannot be combined, a qualitative description is made.

#### 2.5.9 Subgroup analysis

If heterogeneity is significant, we will use a subgroup analysis to determine the source of heterogeneity, including sample size, age, gender, disease severity, type, dose, dosage form, and course of treatment.

#### 2.5.10 Sensitivity analysis

We will conduct sensitivity analysis as comprehensively as possible, by eliminating studies with high risk of bias, missing data studies and outliers to determine whether the conclusion is robust or not.

#### 2.5.11 Grading the quality of evidence

We will use GRADE (The Grading of Recommendations Assessment Development and Evaluation) to assess the quality of systematic review.

## 3 Discussion

Insomnia has become a common and frequently-occurring disease in today's society. It not only seriously affects people's quality of life, but also people suffer from mental illness,^[15]^ Alzheimer disease,^[16]^ cardiovascular events,^[17–19]^ etc. The risk factors of insomnia, which seriously jeopardize the growth of adolescents,^[20,21]^ have caused a heavy social and economic burden^[22]^ and have became a global public health problem.^[4]^ The dominating therapies for insomnia in modern medicine is still drug treatment, but the long-term application of drug treatment has potential risks such as adverse reactions and addiction. In addition, the prognosis of the disease is not optimistic. Traditional Chinese medicine has had a profound insight of insomnia more than 2000 years ago. Xiaoyao san has been widely used in the treatment of insomnia in China. Currently, Xiaoyao has been used to treat various insomnia such as senile insomnia, adolescent insomnia type sub-health, menopausal women insomnia,^[23]^ chronic hepatitis B combined with insomnia,^[24]^ insomnia with anxiety and depression^[25]^, type II diabetes with insomnia^[26]^ and other applications have been reported in the literature, and some people have inferred Xiaoyao san through animal experiments. The mechanism of treatment of psychological stress insomnia may be achieved by changing the content of neurotransmitters such as serotonin, norepinephrine, substance P, and repairing damage of tissue cells caused by neuroendocrine disorders.^[27]^ However, the effectiveness and safety of Xiaoyao san in the treatment of insomnia is still uncertain. Therefore, it is necessary to conduct a high quality systematic review and meta-analysis, in which our rigorous approach will provide objective evidence for Xiaoyao san treatment for insomnia. However, such as some potential shortcomings in this systematic review, there may be a heterogeneity risk due to different nationalities, different doses of herbs, and the age of the patient and the small sample.

## Author contributions

**Conceptualization:** Jiamin Li, Zhenni Mu.

**Data curation:** Tongtong Ma, Ying Zhang.

**Formal analysis:** Tongtong Ma, Ying Zhang.

**Methodology:** Jing Teng.

**Project administration:** Xianghua Qi.

**Supervision:** Jing Teng.

**Validation:** Jiamin Li, Zhenni Mu, Xianghua Qi.

**Writing – original draft:** Jiamin Li, Zhenni Mu, Jun Xie.
